# Elucidation of the mechanism of Zhenbao pills for the treatment of spinal cord injury by network pharmacology and molecular docking: A review

**DOI:** 10.1097/MD.0000000000036970

**Published:** 2024-02-16

**Authors:** Mengru Xu, Wenwen Zhang, Sheng Xu, Xiaochen Niu, Li Wang, Xiaohui Wang, Haihu Hao

**Affiliations:** a Department of Orthopedics, Shanxi Bethune Hospital, Shanxi Academy of Medical Sciences, Third Hospital of Shanxi Medical University, Tongji Shanxi Hospital, Taiyuan, Shanxi, China; b First Clinical Medical College, Shanxi University of Chinese Medicine, Jinzhong, Shanxi, China; c Periodical Press of Fujian Journal of TCM, Fujian University of traditional Chinese Medicine, Fuzhou, Fujian, China; d Basic Medical Research Center, Shanxi Medical University, Taiyuan, Shanxi, China; e Fifth Clinical Medical College, Shanxi Medical University, Taiyuan, Shanxi, China.

**Keywords:** ethnopharmacology, molecular docking simulation, network pharmacology, signal pathways, spinal cord injuries, Zhenbao pill

## Abstract

To explore the mechanism of the Zhenbao pill (ZBP) in treating spinal cord injury (SCI). The TCMSP Database, HERB Database and literature search were used to screen the effective ingredients and targets of ZBP; SCI-related genes were searched in GeneCards, OMIM, PharmGkb, TTD and DrugBank databases; the potential targets of ZBP for treating SCI were predicted and Venn diagrams were drawn, and the “herb-ingredient-target” network was constructed by Cytoscape software. The PPI network was constructed by STRING software, and the core targets were screened by cytoNCA plug-in; GO enrichment and KEGG pathway analysis were performed on the predicted targets using the DAVID Platform, and visualized with the Microbiology Network Platform. The molecular docking between the key ingredients and the core target was carried out by AutoDockVina software. 391 active ingredients and 836 action targets were obtained from ZBP and there are 1557 SCI related genes in 5 disease databases. The top 5 active ingredients were Quercetin, Camptothecin, Kaempferol, Ethyl iso-allocholate, and Ethyl linoleate, and 5 core genes were SRC, CTNNB1, TP53, AKT1, and STAT3. GO enrichment analysis showed that the core targets were involved in 1206 biological processes, 120 cellular components and 160 molecular functions; KEGG enrichment analysis showed that the core targets involved 183 pathways, including PI3K-Akt signaling pathway and other signaling pathways. Molecular docking indicated that CTNNB1, SRC, TP53, AKT1 and STAT3 showed good binding ability with the active ingredients quercetin, kaempferol and ethyl isobutyric acid. ZBP improves SCI through multi-components, multi-targets and multi-pathways.

## 1. Introduction

Spinal cord injury is a disabling disease that causes damage to the corresponding segments of the spinal cord due to various factors and is divided into primary and secondary injuries. Primary injury initiates a complex cascade of secondary injury responses, which cause neuronal and glial cell death, local tissue ischemia and inflammation.^[1]^ The current clinical treatment for the early stages of spinal cord injury is still mainly surgical combined with high-dose MP (Methylprednisolone, MP). Although it can prolong the life of patients to some extent, its side effects cannot be ignored.^[2]^ With further research, it has been found that MP does not promote the recovery of neurological damage in spinal cord injury patients^[3]^ therefore, the use of high-dose MP for spinal cord injury remains controversial. With the continuous development of Chinese medicine, the use of herbal medicine for spinal cord injury has also become a focus of scholarly attention.^[4]^

Zhenbao pill (ZBP) is a kind of Mongolian medicine, which consists of 29 kinds of medicines. And it has the effect of clearing heat, calming the mind and soothing the meridians and activating the channels, and can be used to treat white vein disease. White vein disease means dysfunction or pathological damage of the nervous system. Some studies have shown that Zhen bao pill can repair damaged nerve cells, scavenge oxygen free radicals,^[5]^ has a protective effect on nerves after spinal cord injury, and can promote the recovery of motor function in rats with spinal cord injury.^[6]^ However, the mechanism of action of ZBP as a compounding agent for the treatment of spinal cord injury (SCI) is not clear. Therefore, the innovation of this paper is that we cited the theory of “network pharmacology” proposed by pharmacologist Andrew L. Hopkins^[7]^and the theory of “correlation between Chinese medicine and biomolecular network” proposed by TCM expert Prof Li,^[8]^ as well as the molecular docking method to conduct a preliminary investigation on the mechanism of action of Zhenbao pill in the treatment of spinal cord injury, to provide a scientific basis for its clinical application.

## 2. Materials and methods

### 2.1. Obtain the active ingredients and targets of Zhenbao pill

Zhenbao pill was composed of 29 kinds of traditional Chinese medicines(TCMs), including Gan cao, Rou gui, Cao guo ren, Nigella Seeds, She xiang, and so on. Then we entered the names of these 29 TCMs, such as “Gan cao,” into the TCMSP database(https://tcmspw.com/tcmsp.php),^[9]^ and set oral bioavailability ≥ 30% and drug-likeness ≥ 0.18,^[10]^ to retrieve the active ingredients and their targets contained in each traditional Chinese medicine. The HERB database (http://herb.ac.cn)^[11]^ was used for the supplementation of active ingredients of Traditional Chinese medicine,which were not included in the TCMSP database. Some special herbal medicines like “Nigella Seeds” obtained their active ingredients through literature searches, and the Swiss Targets Prediction (http://www.swisstargetprediction.ch/)^[12]^ online platform was used for predicting the targets of ingredients (Possibility > 0.1).

### 2.2. Access to spinal cord injury-related targets

Five disease databases included GeneCards (http://www.genecards.org/),^[13,14]^ OMIM (http://omim.org/),^[15]^ PharmGkb (http://www.pharmgkb.org/),^[16]^ TTD (http://db.idrblab.net/ ttd),^[17]^ and DrugBank (https://go.drugbank.com/)^[18]^were used for searching for disease-related genes with the keyword “spinal cord injury.” The target genes with a relevance score > 10 were included in the GeneCards database, and the disease-related genes obtained from each database were aggregated and de-duplicated to obtain spinal cord injury-related disease targets.

### 2.3. Construction of “herbal-ingredient-target” network and screening of active ingredients

The targets of Zhenbao pill and spinal cord injury-related genes were taken to intersect, and the venn diagram was drawn using the basic version of Xiantao academic bio-information tool (https://www.xiantao.love/products),^[15]^ that is, the potential targets of Zhenbao pill for the treatment of spinal cord injury were obtained. The Cytoscape 3.8.2 software was used to construct the “herbal-ingredient-target” network, and the active ingredients whose effects work were screened according to the Betweenness algorithm. The Cytoscape software was used to correlate herbal medicines, active ingredients, and potential targets, construct an interaction network diagram between drugs and targets, and analyze the main active ingredients of Zhenbao pill according to the Betweenness algorithm. The betweenness centrality (BC) is a common topological parameter for assessing the centrality properties of nodes in a network.^[19,20]^

### 2.4. Acquisition of PPI protein interaction network and core genes

The potential targets of Zhenbao pill for spinal cord injury were entered into the STRING database (https://stringdb.org/),^[21]^ the genus was set to “Homo sapiens,” the confidence level was set to “highest confidence (0.9) “The protein-protein interaction (PPI) network was obtained by hiding the disconnected nodes in the network, and then the nodes with higher scores in the network were extracted by CytoNCA,^[22]^ a plug-in in Cytoscape 3. 8. 2 software, and the nodes with the highest Betweenness scores were used as the key targets for the follow-up study.

### 2.5. GO function and KEGG pathway enrichment analysis

Gene Ontology (GO) annotation and Kyoto Encyclopedia of Genes and Genomes (KEGG) pathway enrichment analysis^[23,24]^ were performed using the online platform David database (https://david.ncifcrf.gov/).^[25,26]^ GO categories comprise biological processes (BP), cellular components, and molecular functions. The data were ranked according to GO-term, Count, and *P* value, and those with *P* < .05 were screened for inclusion in the statistics, and the top 10 were selected for GO function and the top 30 for KEGG were selected for visualization and analysis in an online platform (http://www.bioinformatics.com.cn).^[27]^

### 2.6. Molecular docking analysis

**Figure 1.**
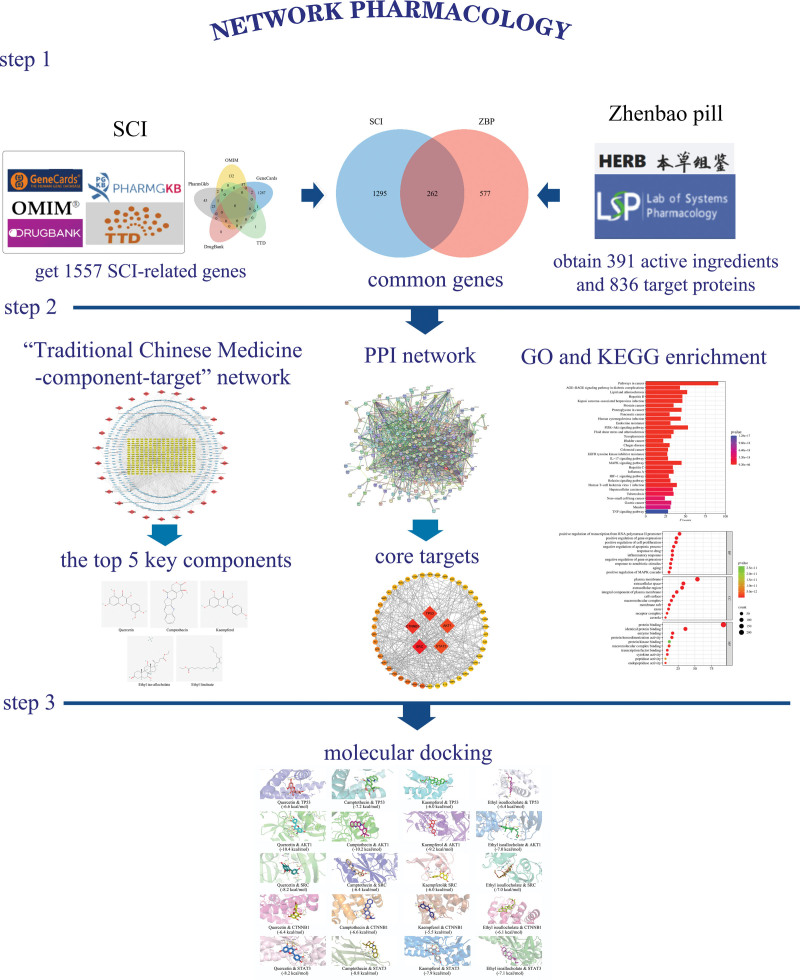
Flow chart of network pharmacology.

The molecular docking technique uses computers to simulate intermolecular interaction relationships through spatial recognition and energy recognition between molecules. In this study, 2D files of small molecule ligands were downloaded by querying the PubChem database (https://pubchem.ncbi.nlm.nih.gov/),^[28]^ and the ligand formats were optimized and converted to 3D structures by ChemBio3D 14.0.0 software.The PDB format files of the receptors were obtained by downloading from the Uniprot database (https://www.uniprot.org/) and the PDB database (http://www1.rcsb.org/), and the receptors were de-hydrogenated and de-residualized using PyMOL 2.4 software (https://pymol.org/2/).^[29–31]^ Use AutoDockTools 1. 5. 6 software (http://autodock.scripps.edu/)^[15]^ for hydrogenation, charge calculation, etc. of small molecule ligands and receptors, and debug the size of the active pocket. Docking was performed using the Vina open source program (http://vina.scripps.edu/),^[16]^ and the binding strength and activity of the target and the active compound were evaluated based on the Docking Score value, and docking was considered feasible if the binding energy was less than −5 kcal/mol,^[32]^ and finally visualized by Pymol software (see Fig. 1, which shows the methods and processes of network pharmacology).

## 3. Results

### 3.1. Active ingredients and targets screening of Zhenbao pill

The chemical ingredients of each traditional Chinese medicine were retrieved from the TCMSP database and screened for active ingredients satisfying oral bioavailability ≥ 30% and drug-likeness  ≥ 0.18. Among them, Fang hai and Shi gao belonged to the mineral category, and the main components were calcium carbonate and calcium phosphate. Therefore, the corresponding targets of them were not found in the database. The active ingredients of Nigella Seeds were 55 species according to the literature search supplement.^[33,34]^The other traditional Chinese medicines and the number of their active ingredients and targets in Zhenbao pills screened from the TCMSP and HERB databases were listed in the Table (see Table 1, which demonstrates the number of active ingredients and targets in Zhenbao pills). By deleting the duplicate items, 391 active ingredients and 836 target proteins were obtained (see Table S1, Supplemental Digital Content, http://links.lww.com/MD/L495 which illustrates all active ingredients and targets of Zhenbao Pill).

**Table 1:** The number of active components and action targets in Zhenbao pills.

Herbs	Active components	Action targets
Gan cao	76	204
Rou gui	75	174
Cao guo ren	69	290
Nigella Seeds	55	323
She xiang	51	160
Feng xiang zhi	33	260
Bai dou kou	29	136
Jiang xiang	25	63
Bai ju sheng	20	249
Hong hua	16	193
Tu mu xiang	14	47
Zhi zi	12	177
Jue ming zi	12	55
Bi ba	11	60
Zhen zhu	9	0
Rou dou kou	8	54
Cheng xiang	8	163
He zi	7	67
Hai jin sha	7	82
Ding xiang	6	168
Chuan lian zi	6	140
Qing ma zi	6	30
Shui niu jiao	6	68
Mu xiang	5	28
Di jin cao	5	154
Niu huang	4	5
Tan xiang	3	73
Fang hai	2	0
Shi gao	1	0

### 3.2. Related targets of spinal cord injury

**Figure 2.**
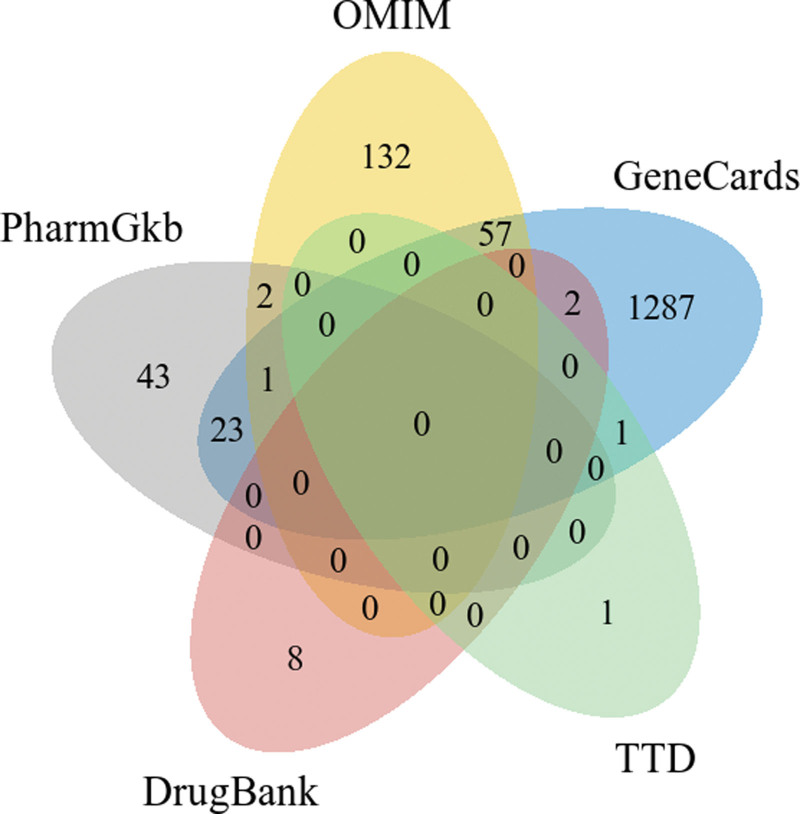
Spinal cord injury-related targets.

Based on the keyword “spinal cord injury” and the filtering criteria of a relevance score > 10, we retrieved 1371, 192, 69, 7, 10 disease targets in 5 disease databases, namely, GeneCards, OMIM, PharmGkb, TTD and DrugBank, respectively. And then we integrated these targets and removed duplicate values to obtain a total of 1557 SCI-related targets (see Fig. 2, and Table S2, Supplemental Digital Content, http://links.lww.com/MD/L497 which show the SCI-related targets searched from the 5 databases above).

### 3.3. Predicting the potential targets of Zhenbao pill for spinal cord injury and constructing an “herbal-ingredient-target” network

**Figure 3.**
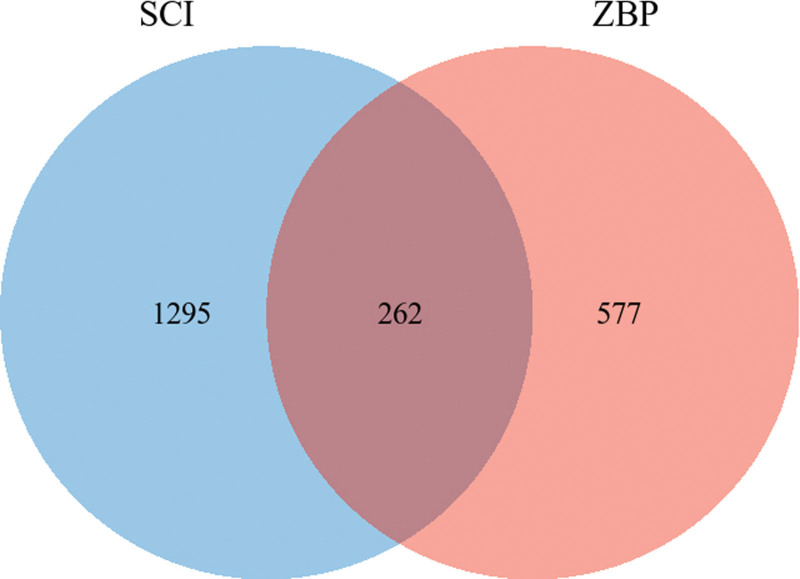
Targets prediction of Zhenbao pill for spinal cord injury.

**Figure 4.**
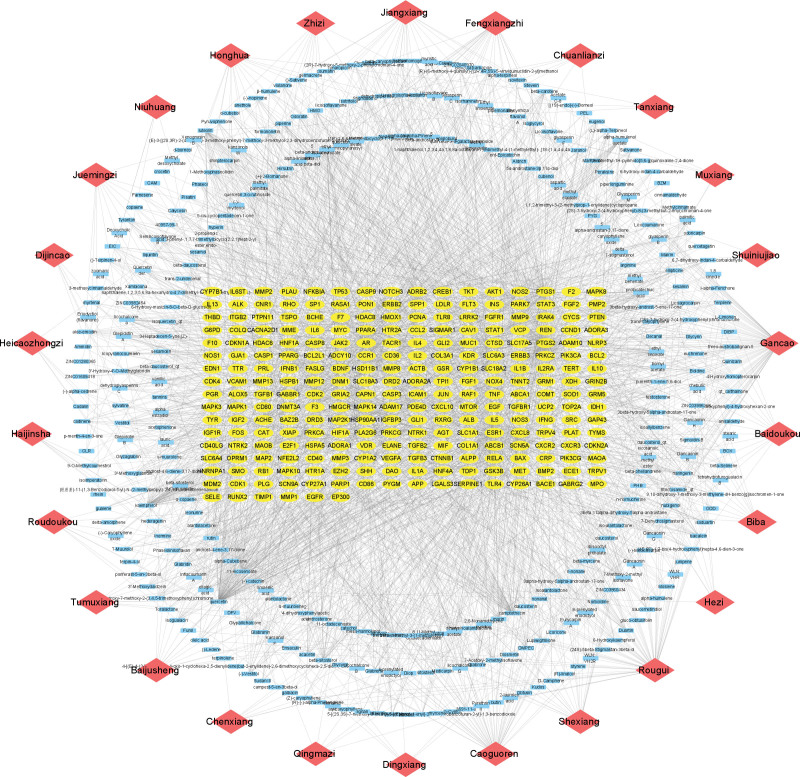
Construction of “herbal-ingredient-target” network.

**Figure 5.**
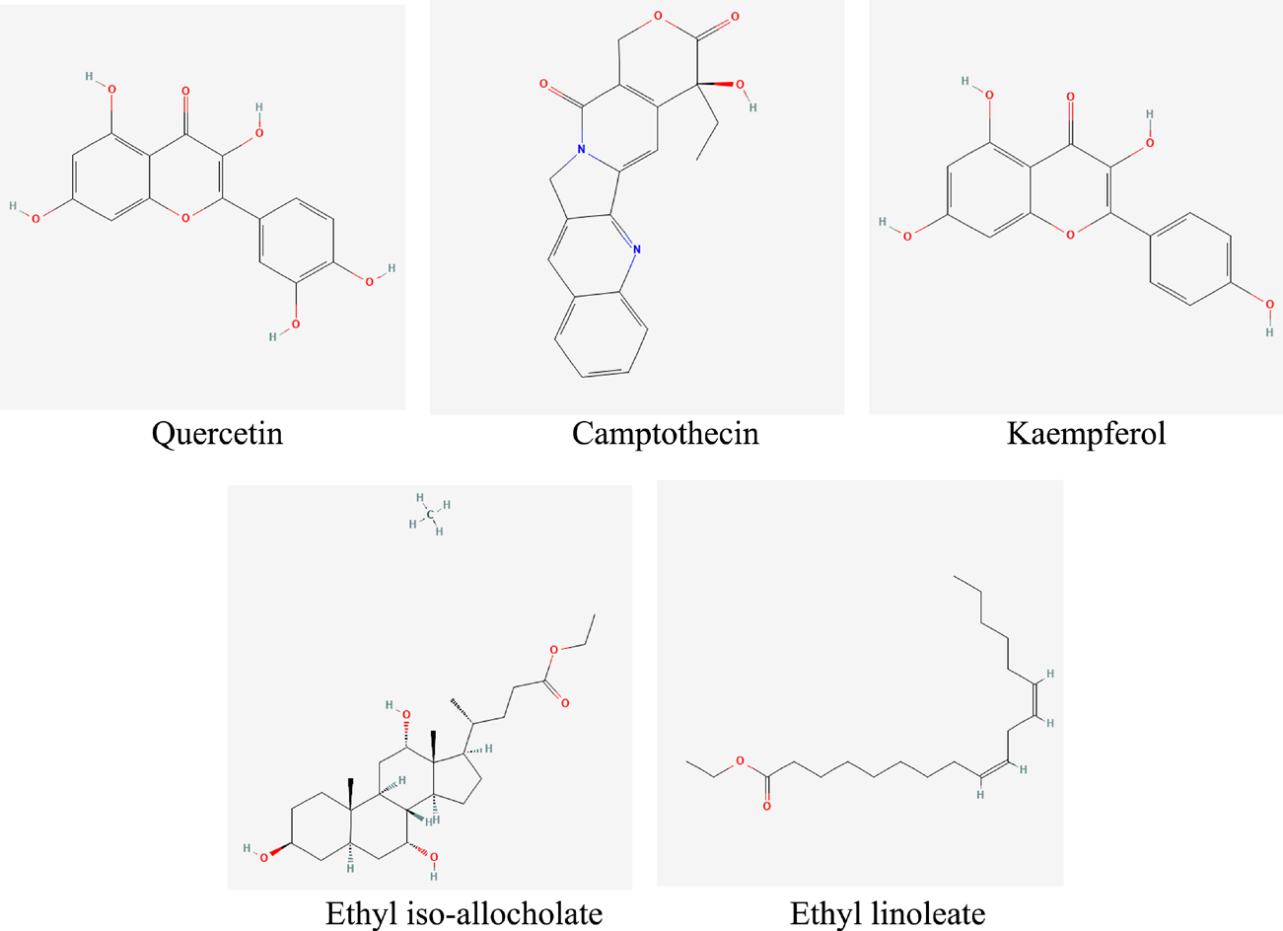
Chemical structure formulas of the top 5 active ingredients.

The targets of the active ingredients were intersected with the genes related to spinal cord injury, and 262 potential targets were obtained, and the venn diagram was drawn on the online platform of Xiantao academic biological information tool (see Fig. 3, which demonstrates the number of common targets of SCI and ZBP; see Table S3, Supplemental Digital Content, http://links.lww.com/MD/L500 which illustrates all common targets of SCI and ZBP). The network of “herbal-ingredient-target” was constructed (see Fig. 4, which shows the network relationship between herbals, ingredients, and targets), and the top 5 active ingredients were screened: Quercetin, Camptothecin, Kaempferol, Ethyl iso-allocholate, and Ethyl linoleate (see Fig. 5 and Table 2, which illustrates network topological parameters of the top 5 active ingredients).

**Table 2:** Network topological parameters of key targets.

ID	Active ingredient	Betweenness centrality
MOL000098	Quercetin	70159.94
HBIN019519	Camptothecin	27517.594
MOL000422	Kaempferol	19916.045
HBIN025933	Ethyl iso-allocholate	19016.723
HBIN025942	Ethyl linoleate	15279.536

### 3.4. Establishment of PPI networks and screening of core targets

**Figure 6.**
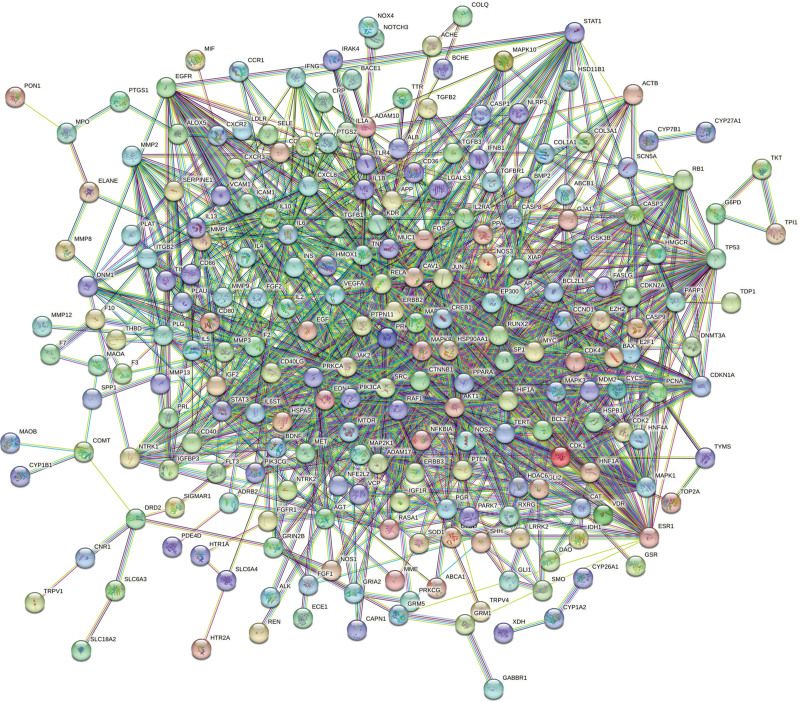
The PPI network of target proteins.

**Figure 7.**
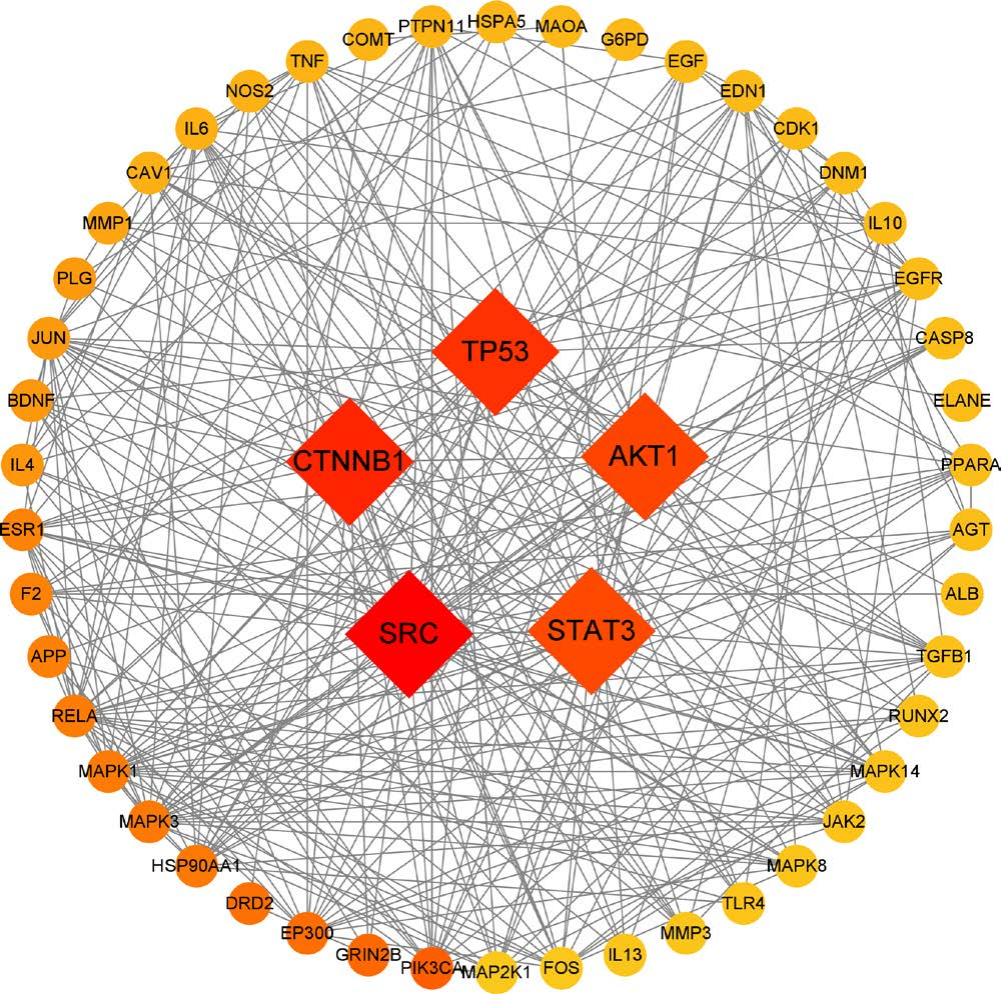
Core targets screening.

The potential targets were imported into the STRING network analysis platform to obtain the PPI protein interactions map (see Fig. 6, which shows the protein interactions between potential targets). The core genes were screened using the cytoNCA plugin (see Fig. 7, which shows core genes of Zhenbao pills in the treatment of SCI), and 5 core genes were obtained: SRC, CTNNB1, TP53, AKT1, and STAT3 (see Table 3, which illustrates the betweenness centrality of the top 5 genes).

**Table 3:** Potential targets of Zhenbao pills in the treatment of SCI.

Uniprot ID	PBD ID	Gene symbol	Protein name	Betweenness centrality
P12931	1A07	SRC	Proto-oncogene tyrosine-protein kinase Src	5001.0156
P35222	7AFW	CTNNB1	Catenin beta-1	4094.1108
P04637	1C26	TP53	Cellular tumor antigen p53	3796.4304
P31749	4EJN	AKT1	RAC-alpha serine/threonine-protein kinase	3333.877
P40763	6NUQ	STAT3	Signal transducer and activator of transcription 3	3202.2354

### 3.5. GO function and KEGG pathway enrichment analysis

**Figure 8.**
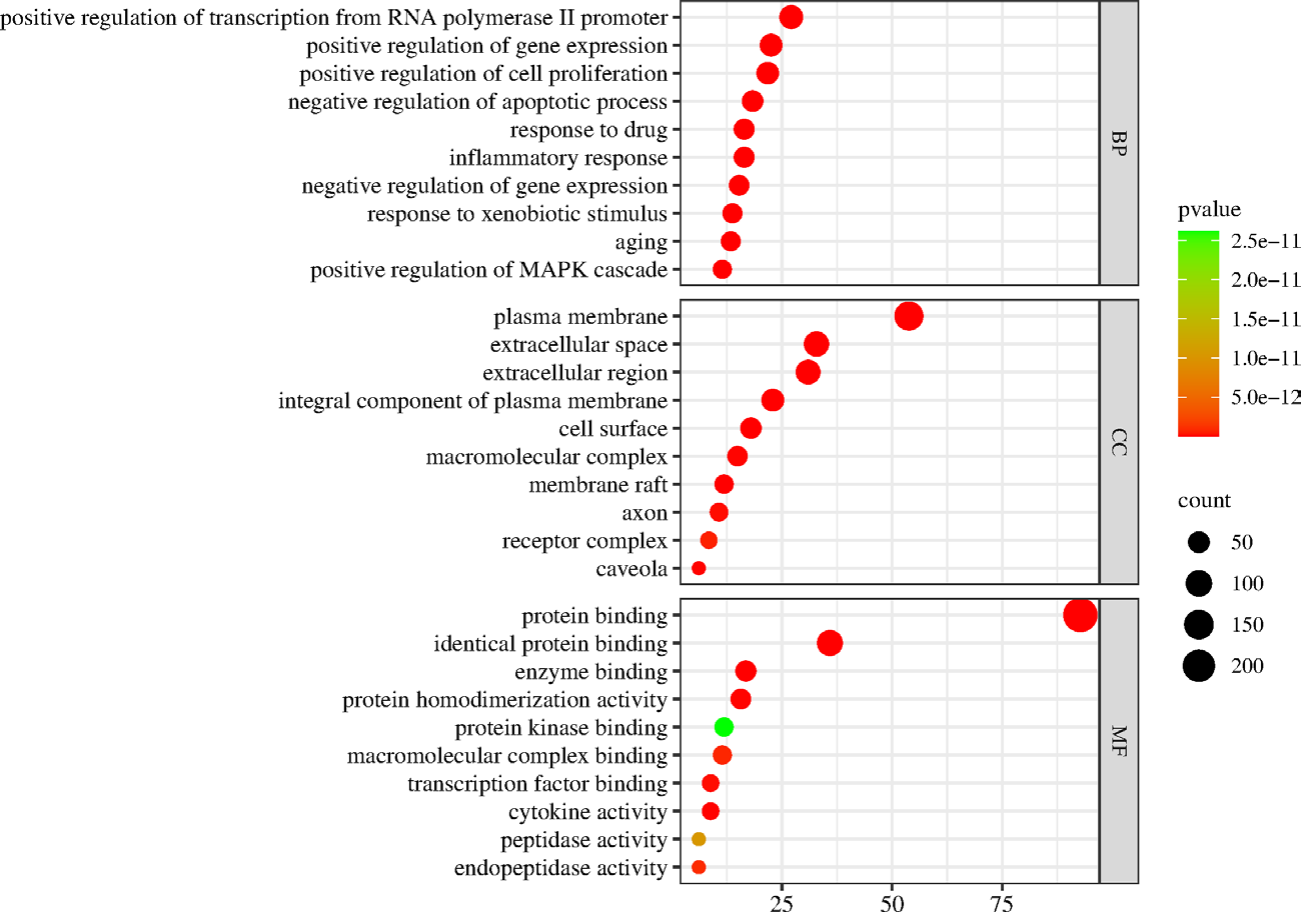
GO function enrichment analysis.

**Figure 9.**
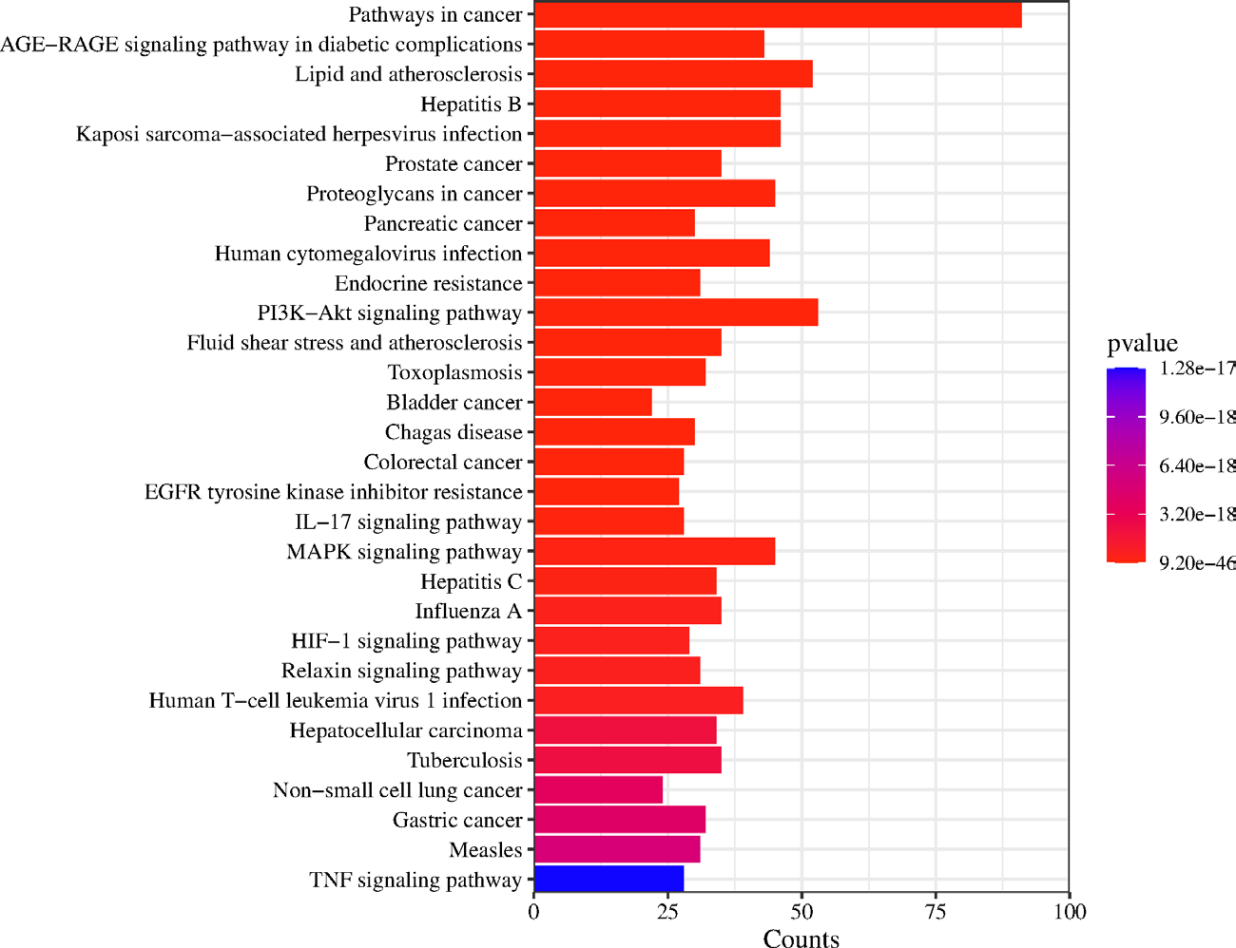
KEGG pathway enrichment analysis.

The 262 predicted targets were imported into the Database of Annotated Biological Information database for GO function and KEGG pathway enrichment analysis, and 640 GO entries and 183 KEGG pathways were obtained with *P* < .05 as the filtering condition. The GO function includes 1206 entries of BP, 120 entries of cellular component, and 160 entries of molecular function. After sorting according to the *P* value, the top 10 entries are selected to draw a bubble chart. For the KEGG pathways, select the top 30 entries to draw a bar chart (see Figs. 8 and 9, which show the visualization of partial results of GO function and KEGG pathway enrichment analysis, see Tables S4 and S5, Supplemental Digital Content, http://links.lww.com/MD/L505, http://links.lww.com/MD/L506 which demonstrates the results of GO function and KEGG pathway enrichment analysis).

GO enrichment results showed that in biological processes, crossover genes are mainly associated with cell proliferation, transcriptional regulation, inflammatory response, apoptosis, signaling pathways, response to drugs and foreign biological stimuli, positive regulation of MAPK cascade response and other biological processes; cellular component enrichment results showed that crossover genes are widely distributed in extracellular, cytoplasmic, cell membrane, neuronal cell axons and other sites; molecular function enrichment The results showed that the crosslinked genes could affect protein binding, enzyme binding, transcription factor activity, protein kinase binding, peptidase activity, homodimer activity, etc.

KEGG pathway enrichment analysis showed that the signaling pathways involved in the intersection genes include cancer-related pathways such as prostate cancer, pancreatic cancer, bladder cancer colorectal cancer, hepatocellular carcinoma, tuberculosis, non-small cell lung cancer, gastric cancer; in addition, there are infectious disease-related pathways such as influenza A, hepatitis B, hepatitis C, human T-cell leukemia virus type 1 infection, human cytomegalovirus infection; and involved in PI3K-Akt signaling pathway, IL-17 signaling pathway, MAPK signaling pathway, HIF-1 signaling pathway, MAPK signaling pathway, TNF signaling pathway and other signaling pathways, indicating that Zhenbao pill has multi-ingredient, multi-target and multi-pathway effects.

### 3.6. Molecular docking analysis

**Figure 10.**
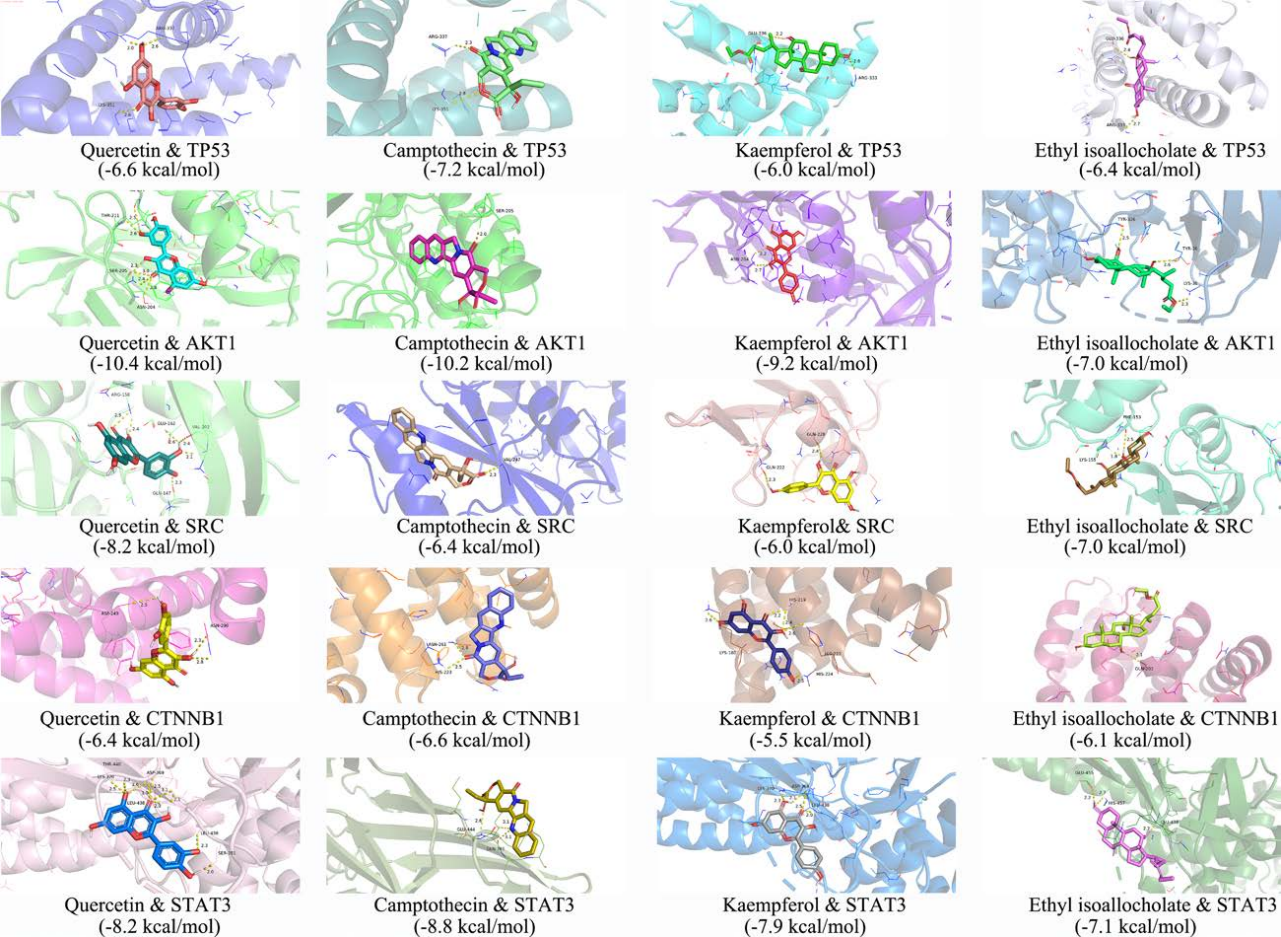
Molecular docking visualization.

The 5 main active ingredients in Zhenbao pill, quercetin, camptothecin, kaempferol, ethyl isobutyrate, and ethyl linoleate, were simulated to dock with the key core targets TP53, AKT, SRC, CTNNB1, and STAT3, respectively. Binding energies less than −5 kcal/mol are generally considered to indicate meaningful docking results (see Table 4, which shows the docking and binding ability of main active ingredients to core targets). Among them, the binding energy of ethyl linoleate to the 5 major targets was greater than -5kcal/mol, indicating a poor binding effect.^[35]^ The docking results with good binding energy were visualized and analyzed (see Fig. 10, which shows the visualization of molecular docking).

**Table 4:** Docking and binding ability of main active ingredients to core targets.

Target	Binding energy (kcal/mol)				
	Quercetin	Camptothecin	Kaempferol	Ethyl iso-allocholate	Ethyl linoleate
TP53	−6.6	−7.2	−6.0	−6.4	−2.7
AKT1	−10.4	−10.2	−9.2	−7.0	−3.6
SRC	−8.2	−6.4	−6.0	−7.0	−2.9
CTNNB1	−6.4	−6.6	−5.5	−6.1	−3.1
STAT3	−8.2	−8.8	−7.9	−7.1	−2.7

## 4. Discussions

Chinese medicine is widely used in scientific research and clinical practice related to spinal cord injury due to its advantages of holistic regulation, multi-target superiority and low toxic side effects.^[2,4,36]^ Due to the complexity of its composition and compounding, Traditional Chinese medicine compound prescription has existed as empirical medicine in the clinic, which cannot be assessed quantitatively. With the rapid development of multidisciplinary approaches such as bioinformatics, systems biology and pharmacology, a new interdisciplinary approach has been proposed, namely, network pharmacology.^[7,37]^ The network pharmacology approach is based on the “compound-protein/gene-disease” network pathway, which systematically reveals the complexity and correlation between biological systems, drugs, and diseases, which is consistent with the holistic view of Chinese medicine, and is more comprehensive and systematic for the pharmacological study of compounded drugs.^[38]^The Mongolian medicine Zhenbao pill (Erden-Urile) is effective in the treatment of patients with neurological disorders such as stroke, hemiplegic sequelae and cerebral edema caused by incomplete cerebral ischemia/reperfusion.^[39,40]^ Clinical treatment with the aid of Zhenbao pill is apparently more effective than conventional treatment alone in treating patients with cerebral infarction, which largely confirms the efficacy of Zhenbao pill in relieving cardiovascular spasm, improving blood circulation and increasing blood supply to brain tissue cells.^[41]^ In addition, Zhenbao pill is effective in promoting the recovery of motor function in rats with spinal cord injury,^[6]^ however, the exact mechanism of action in spinal cord injury is not yet clear.

The network pharmacological analysis showed that the main active ingredients of Zhenbao pill are quercetin, kaempferol, ethyl isobutyric acid, α-tigerol acetate and ethyl linoleate. GO enrichment results indicated that the target genes regulated by Zhenbao pill may be distributed in cell membrane, neuronal axon, cytoplasm and extracellular, which are involved in regulating cell inflammation, cell proliferation and apoptosis, etc. The KEGG pathway analysis showed that Zhenbao pill is involved in regulating KEGG pathway analysis showed that Zhenbao pill is involved in regulating PI3K-Akt signaling pathway, IL-17 signaling pathway, HIF-1 signaling pathway and MAPK signaling pathway.

AKT1 is a member of the serine/threonine protein kinase family (AKT), and the PI3K/Akt pathway is closely associated with pro- and anti-inflammatory responses. The Akt signaling pathway can regulate the activity, including inflammatory cytokines and macrophage phagocytosis.^[42,43]^ It has been shown that quercetin can activate PI3K/Akt signaling pathway-induced autophagy in rat neurons to attenuate the inflammatory response after spinal cord injury.^[44]^Interleukin 17 (IL-17), a pro-inflammatory cytokine, may promote the activation of STAT3 signaling pathway by upregulating the expression of IL-6, IL-21 and IL-23 cytokines in a rat model of acute spinal cord injury, thereby mediating the upregulation of IL-17 cytokine expression, and the 3 may form a cycle that interacts and promotes each other to participate in acute inflammatory response secondary to spinal cord injury.^[45]^ Hypoxia-inducible factor 1 (HIF-1) is an oxygen-regulated transcriptional activator. hIF-1 includes HIF1-α and HIF1-β. HIF1-α is expressed cumulatively under hypoxic conditions, whereas HIF1-β is expressed constitutively. Under hypoxic conditions, p53 can directly interact with HIF1-α and inhibit hypoxia-induced HIF1-α expression by promoting MDM2-mediated ubiquitination and proteasomal degradation.^[46]^ MAPK can be divided into 4 subfamilies: ERK, p38, JNK and ERK5, with the p38/MAPK pathway primarily transducing inflammatory cytokines and multiple types of cellular stress signals. In rats with spinal cord injury, quercetin exerts a protective effect on the spinal cord by inhibiting p38MAPK/iNOS signaling pathway activation, decreasing nitric oxide synthase, increasing superoxide dismutase, and inhibiting oxidative stress^[47]^; the protective effect of kaempferol on neurotoxin 6-hydroxydopamine (6-OHDA)-induced inflammatory injury in PC12 cells is also associated with its inhibition of the p38/MAPK pathway.^[48]^ In addition, kaempferol pretreatment attenuates microglia-mediated neuroinflammation by inhibiting MAPKs-NF-κB signaling pathway and focal death secondary to spinal cord injury.^[49]^ Some studies have shown that ethyl isobutyrate induces apoptosis and reduces tumor growth, metastasis and angiogenesis in vivo in cancer cells and is safe for normal tissues,^[50]^ Camptothecin can inhibit pyroptosis, control neuroinflammation, and improve functional recovery after SCI.^[51]^ But there are fewer studies on the mechanism of these drugs.

Molecular docking results suggest that the main active ingredients quercetin, kaempferol and ethyl isobutyrate have good docking ability with target proteins TPT53, AKT1, SRC, CTNNB1. p53 is a tumor protein encoded by TP53, which can mediate cell proliferation, apoptosis, inflammatory response, autophagy, etc.,^[52]^ kaempferol treatment can induce upregulation of p53, Bad and Bax proteins thus induce apoptosis in cancer cells.^[53]^ The expression level of p53 is elevated after spinal cord injury,^[54]^ and pharmacological intervention to reduce p53 can slow down the inflammatory response in rats.^[55]^ CTNNB1 (Catenin beta-1), also known as beta-catenin, is widely studied to alleviate secondary spinal cord injury by activating the Wnt/beta-catenin signaling pathway, which is the most classical of the Wnt signaling pathway, in which beta-catenin is the most important ingredient of this pathway. When the Wnt/βcatenin signaling pathway is activated, free β-catenin increases in the cytoplasm, which can enter the nucleus and interact with transcription factors to promote the transcription of Wnt pathway target genes and the expression of related proteins, causing cell proliferation, vascular repair and neuronal axon regeneration.^[56]^ STAT3 is a member of the STAT family of proteins that respond to cytokines and growth factors, and when STAT3 is phosphorylated, it forms homodimers or heterodimers and translocates to the nucleus to function as a transcription factor. It has been reported that early pathological changes in spinal cord injury may involve inflammatory responses of neuronal cells, autophagy, apoptosis and activation of the JAK2/STAT3 signaling pathway. Targeted intervention in the activation of the JAK2/STAT3 signaling pathway may inhibit the process of spinal cord injury and thus provide an experimental basis for targeted therapy of spinal cord injury.^[57]^ Src is a non-receptor tyrosine kinase protein that in humans is encoded by the SRC gene It is encoded by the SRC gene in humans. Phosphorylation of ephexin1 by Src kinase after spinal cord injury may be involved in the collapse of axon growth cones and inhibit axon generation, whereas the Src kinase inhibitor PP2 restores functional movement, increases preserved white matter tissue and stimulates axon growth in rats with moderate spinal cord injury.^[58]^ It can be seen that the core targets TP53, AKT1, SRC, and CTNNB1 all play important roles in the development of spinal cord injury, and how quercetin, kaempferol, and ethyl isobutyrocholate, which have good docking results with the core targets, regulate the expression of these proteins will be further verified by experiments. In conclusion, the network pharmacology provides some ideas for the specific mechanism of Zhenbao pill in treating spinal cord injury.

## 5. Conclusions

In conclusion, the treatment of spinal cord injury with Zhenbao pill is a multi-ingredient, multi-target and multi-pathway synergistic process. The results suggest that the active ingredients in this compound, such as quercetin, kaempferol, and ethyl isobutyrate, may act on target proteins such as TPT53, AKT1, SRC, and CTNNB1, regulate IL-7, PI3K-Akt, MAPK, and HIF-1 pathways, and exert their effects on improving spinal cord injury through anti-apoptosis, inhibition of oxidative stress, and suppression of inflammatory response. However, the exact mechanism should be verified via animal or clinical studies and we will continue to explore in the follow-up study.

## Author contributions

**Conceptualization:** Li Wang.

**Funding acquisition:** Haihu Hao.

**Investigation:** Mengru Xu, Xiaochen Niu, Haihu Hao.

**Methodology:** Mengru Xu.

**Resources:** Wenwen Zhang, Sheng Xu.

**Software:** Mengru Xu, Xiaochen Niu.

**Supervision:** Xiaohui Wang.

**Visualization:** Mengru Xu, Wenwen Zhang, Sheng Xu.

**Writing – original draft:** Mengru Xu.

**Writing – review & editing:** Li Wang, Xiaohui Wang, Haihu Hao.

## Supplementary Material










