# Supplementary material for: Elucidation of the mechanism of Zhenbao pills for the treatment of spinal cord injury by network pharmacology and molecular docking: A review
Source: Medicine (Baltimore). 2024 Feb 16;103(7):e36970. doi: 10.1097/MD.0000000000036970 (PMC10869052; doi:10.1097/MD.0000000000036970)
Supplement: Supplementary file 2 [file medi-103-e36970-s002.docx]

Table S2. The SCI-related targets searched from the five disease databases

| Database | Genes |
| --- | --- |
| OMIM (192) | AGRN B3GALT6 MXRA8 SSU72 PRKCZ SKI HES5 ACOT7 PLEKHG5 UTS2 SLC25A33 NMNAT1 KIF1B CORT TARDBP MASP2 MTOR DRAXIN MTHFR CLCN6 PLOD1 MFN2 TNFRSF1B DHRS3 SPEN PADI2 PADI6 DDOST ECE1 HSPG2 EPHA8 C1QA C1QC C1QB EPHB2 KDM1A HNRNPR RPL11 GRHL3 RUNX3 SELENON STMN1 STX12 YARS1 AZIN2 CSMD2 NCDN ADPRS RSPO1 ZMPSTE24 EDN2 RIMKLA ERMAP ELOVL1 SZT2 ARTN BEST4 PTCH2 EIF2B3 HPDL MUTYH MMACHC PRDX1 AKR1A1 POMGNT1 TAL1 STIL ORC1 COA7 CPT2 DHCR24 MYSM1 FGGY NFIA DOCK7 ANGPTL3 DNAJC6 CTH TNNI3K USP33 TTLL7 BTBD8 ABCA4 F3 PLPPR5 DBT WDR47 C1orf194 SORT1 GPR61 SLC6A17 DRAM2 ADORA3 DDX20 SLC16A1 PTPN22 TRIM33 NRAS SYCP1 NGF VANGL1 ATP1A1 PHGDH REG4 NOTCH2 SEC22B TXNIP HYDIN2 SEMA6C TNFAIP8L2 ZNF687 IVL JTB EFNA3 MUC1 GBA PKLR GON4L LMNA SEMA4A BGLAP NAXE NTRK1 PEAR1 AIM2 CRP KCNJ10 VANGL2 MPZ SDHC FCGR2A FCGR2B DDR2 RGS5 LMX1A RXRG ALDH9A1 TMCO1 CREG1 ADCY10 ATP1B1 F5 SELP SELE KIFAP3 PRRC2C MYOC DNM3 FASLG DARS2 TNR COP1 TOR1AIP1 ACBD6 NMNAT2 NCF2 TRMT1L HMCN1 PRG4 PTGS2 PLA2G4A KCNT2 CFH CFHR3 CFHR1 LHX9 PTPRC KIF14 INAVA TMEM9 TNNT2 NAV1 SHISA4 GPR37L1 ADORA1 CHI3L1 ATP2B4 REN PPP1R15B NFASC CNTN2 DSTYK MAPKAPK2 IL10 CD55 CD34 HSD11B1 IRF6 PACC1 NENF FLVCR1 PROX1 |
| TTD (7） | NR3C1 SOD Cu-Zn TLR9 RGMA GRIA ROCK RHO |
| DrugBank(10） | RYR1 PTPRS ATP6V1A SLC25A4 SLC25A5 SLC25A6 CACNA2D1 SLC7A5 SLC1A1 SERPINA7 |
| PharmGkb(69) | DRAXIN MDGA2 NKX6-1 GLI2 NKX6-2 NKX2-2 MDGA1 SHH PDGFD KCNK18 UNCX GSX1 EVX1 HOXB8 ERBB2 ABT1 RAB23 GLI3 SERF1A HOXC10 DRGX TMEM123 NINJ1 GSX2 PAX7 GDF11 PHGDH LBX1 DCC CHRD HAVCR1 SLIT1 OLIG2 HOXD10 TULP3 CACNA1A MNX1 GDF7 ARF4 SLIT3 DPYSL2 LTC4S SUFU RELN SLIT2 ERCC2 GIPR TXN2 SPP1 ROBO1 LGALS1 GIP CLN8 IGHMBP2 MAX AKAP5 DRD3 EPO SOD2 SMO PROX1 MAP1B NF1 PCSK1 MAP2K1 AQP1 APP TP73 SOD1 |
| GeneCards(1371) | CYFIP2 BAX PRKCA ITM2B CDKN3 DCN LGI1 EZH2 BSG COL12A1 MAP3K5 MIR29A RAD21 UBE2L3 NDUFS6 NDUFB3 NDUFAF1 PDE4A CDK6 LIG3 SYNJ2 CTSD HDAC8 BLVRB GCDH TLR10 H3C1 MIR324 DLC1 ABCB7 HNRNPA2B1 GNRH1 ATG5 LUC7L2 CHI3L1 NEU1 PAX7 MT-ATP8 SP1 LMX1B LYST MIR184 HMGCR LRP6 ERCC5 TNFSF4 JPH1 HINT1 FZD9 NFKBIA VDAC1 NDUFA6 NDUFA13 AGER TLR8 ALDH1L1 IGF1R CACNA1B NGLY1 ENO1 CD86 CTNNA3 IKZF1 MYH14 RAC1 EXTL3 XBP1 CBLIF SHMT1 COX8C VCAN NOX4 GHRL SOX4 PRKG1 SERPINA7 MIR143 GDF6 FLI1 CLU DCHS1 HERC2 B9D1 GGT1 COL3A1 CELSR2 DCX CFB TYMP SYNGAP1 MEOX1 RNU4ATAC NDUFB11 NSL1 GLRB ROBO3 SPON1 LRP4 SMARCA2 MTMR2 BACE1 MEGF10 MOBP RPE65 GRM5 TMEM126B TIMMDC1 HECW2 BTD HUWE1 LMNB2 NKX2-5 ZMPSTE24 CGB7 AGTR1 CELSR3 RUNX1 EZR ADAM10 SNORA49 CAPN1 MIR132 CR2 GTF2I MLC1 SLC19A3 SPG21 PYGM TTC21B NEAT1 RTEL1 GFM1 FRZB DDX3X GSK3B ANKRD6 BTK MCOLN1 WNT7B INA PTK7 NDUFB9 NDUFA11 CDH5 AGT TNFRSF13B BLOC1S1 PCMT1 WNT11 SERPINE2 E2F1 WARS1 KRT7 CTSA IL2RB HOXD13 CYBB S100A1 MSTN ADAMTS1 CD14 SLC12A2 SRSF2 THPO DPP6 ADD3 CS ACAP2 ITFG1 TP63 MT3 CFAP410 WDR45 TERC IL6ST VPS13D TTPA CGA TLR7 ADAM17 PDGFA HADHB F11 |
