# Supplementary material for: Elucidation of the mechanism of Zhenbao pills for the treatment of spinal cord injury by network pharmacology and molecular docking: A review
Source: Medicine (Baltimore). 2024 Feb 16;103(7):e36970. doi: 10.1097/MD.0000000000036970 (PMC10869052; doi:10.1097/MD.0000000000036970)
Supplement: Supplementary file 4 [file medi-103-e36970-s004.docx]

| NO. | Term | *P*-value | Count | Type |
| --- | --- | --- | --- | --- |
| GO:0010628 | positive regulation of gene expression | 1.08E-36 | 59 | BP |
| GO:0008284 | positive regulation of cell proliferation | 4.18E-33 | 57 | BP |
| GO:0042493 | response to drug | 7.37E-31 | 43 | BP |
| GO:0007568 | aging | 4.37E-28 | 35 | BP |
| GO:0010629 | negative regulation of gene expression | 1.47E-26 | 40 | BP |
| GO:0045944 | positive regulation of transcription from RNA polymerase II promoter | 2.50E-26 | 71 | BP |
| GO:0009410 | response to xenobiotic stimulus | 4.60E-26 | 36 | BP |
| GO:0043066 | negative regulation of apoptotic process | 2.89E-25 | 48 | BP |
| GO:0006954 | inflammatory response | 7.68E-25 | 43 | BP |
| GO:0043410 | positive regulation of MAPK cascade | 1.63E-24 | 30 | BP |
| GO:0005615 | extracellular space | 1.12E-25 | 86 | CC |
| GO:0005886 | plasma membrane | 4.36E-24 | 141 | CC |
| GO:0009986 | cell surface | 6.21E-22 | 47 | CC |
| GO:0045121 | membrane raft | 1.27E-20 | 31 | CC |
| GO:0005576 | extracellular region | 7.64E-20 | 81 | CC |
| GO:0005887 | integral component of plasma membrane | 4.03E-15 | 60 | CC |
| GO:0005901 | caveola | 4.19E-14 | 16 | CC |
| GO:0032991 | macromolecular complex | 4.26E-14 | 39 | CC |
| GO:0030424 | axon | 1.48E-13 | 28 | CC |
| GO:0043235 | receptor complex | 6.12E-13 | 22 | CC |
| GO:0042802 | identical protein binding | 1.07E-32 | 94 | MF |
| GO:0019899 | enzyme binding | 2.02E-26 | 44 | MF |
| GO:0005515 | protein binding | 2.75E-24 | 243 | MF |
| GO:0005125 | cytokine activity | 2.78E-14 | 23 | MF |
| GO:0042803 | protein homodimerization activity | 4.50E-14 | 41 | MF |
| GO:0008134 | transcription factor binding | 1.63E-13 | 23 | MF |
| GO:0044877 | macromolecular complex binding | 7.72E-13 | 30 | MF |
| GO:0004175 | endopeptidase activity | 9.40E-13 | 16 | MF |
| GO:0008233 | peptidase activity | 1.04E-11 | 16 | MF |
| GO:0019901 | protein kinase binding | 2.62E-11 | 31 | MF |

Table S4. The results of GO function enrichment analysis
