# Supplementary material for: Elucidation of the mechanism of Zhenbao pills for the treatment of spinal cord injury by network pharmacology and molecular docking: A review
Source: Medicine (Baltimore). 2024 Feb 16;103(7):e36970. doi: 10.1097/MD.0000000000036970 (PMC10869052; doi:10.1097/MD.0000000000036970)
Supplement: Supplementary file 5 [file medi-103-e36970-s005.docx]

Table S5. The results of KEGG pathway enrichment analysis

| Term | Term | Count | *P*-value |
| --- | --- | --- | --- |
| hsa05200 | Pathways in cancer | 91 | 9.20E-46 |
| hsa04933 | AGE-RAGE signaling pathway in diabetic complications | 43 | 2.29E-38 |
| hsa05417 | Lipid and atherosclerosis | 52 | 1.92E-32 |
| hsa05161 | Hepatitis B | 46 | 7.51E-32 |
| hsa05167 | Kaposi sarcoma-associated herpesvirus infection | 46 | 3.74E-28 |
| hsa05215 | Prostate cancer | 35 | 5.87E-28 |
| hsa05205 | Proteoglycans in cancer | 45 | 5.01E-26 |
| hsa05212 | Pancreatic cancer | 30 | 3.39E-25 |
| hsa05163 | Human cytomegalovirus infection | 44 | 2.67E-23 |
| hsa01522 | Endocrine resistance | 31 | 8.94E-23 |
| hsa04151 | PI3K-Akt signaling pathway | 53 | 1.41E-22 |
| hsa05418 | Fluid shear stress and atherosclerosis | 35 | 3.50E-22 |
| hsa05145 | Toxoplasmosis | 32 | 4.87E-22 |
| hsa05219 | Bladder cancer | 22 | 7.51E-22 |
| hsa05142 | Chagas disease | 30 | 4.93E-21 |
| hsa05210 | Colorectal cancer | 28 | 6.50E-21 |
| hsa01521 | EGFR tyrosine kinase inhibitor resistance | 27 | 9.11E-21 |
| hsa04657 | IL-17 signaling pathway | 28 | 8.91E-20 |
| hsa04010 | MAPK signaling pathway | 45 | 1.91E-19 |
| hsa05160 | Hepatitis C | 34 | 2.36E-19 |
| hsa05164 | Influenza A | 35 | 4.13E-19 |
| hsa04066 | HIF-1 signaling pathway | 29 | 4.91E-19 |
| hsa04926 | Relaxin signaling pathway | 31 | 5.40E-19 |
| hsa05166 | Human T-cell leukemia virus 1 infection | 39 | 6.58E-19 |
| hsa05225 | Hepatocellular carcinoma | 34 | 2.11E-18 |
| hsa05152 | Tuberculosis | 35 | 2.24E-18 |
| hsa05223 | Non-small cell lung cancer | 24 | 3.70E-18 |
| hsa05226 | Gastric cancer | 32 | 4.26E-18 |
| hsa05162 | Measles | 31 | 5.14E-18 |
| hsa04668 | TNF signaling pathway | 28 | 1.28E-17 |
